# Simulation Analysis of a Sandwich Cantilever Ultrasonic Motor for a Dexterous Prosthetic Hand

**DOI:** 10.3390/mi14122150

**Published:** 2023-11-24

**Authors:** Kai Guo, Jingxin Lu, Hongbo Yang

**Affiliations:** 1 School of Biomedical Engineering (Suzhou), Division of Life Sciences and Medicine, University of Science and Technology of China, Hefei 230026, China; 2 Suzhou Institute of Biomedical Engineering and Technology, Chinese Academy of Sciences, Suzhou 215163, China; 3 School of Mechanical and Electrical Engineering, Changchun University of Science and Technology, Changchun 130022, China

**Keywords:** prosthetic hand, ultrasonic motor, modal analysis

## Abstract

Currently, the driving motor used in a dexterous prosthetic hand is limited by the driving principle, and it has the characteristics of a complex structure, slow response, low positioning accuracy, and excessive volume. There are special requirements in terms of quality and quality, and traditional motor drives have greatly affected the progress of prosthetic robots. A motor (ultrasonic motor) has been developed over more than 30 years. It has the advantages of a small size, small mass, simple structure, accurate positioning, high power density, and fast response time, which is enough to improve the driving mechanism performance of the prosthetic hand with a connecting rod. In this paper, the structural characteristics of the prosthetic hand will be analyzed, and the modal analysis, harmonic response analysis, and transient analysis simulation of the longitudinal vibration linear motor stator used in the prosthetic hand with a connecting rod will be carried out in order to provide preliminary preparation for the feasible design and manufacture of the size of the ultrasonic driver structure used for the prosthetic hand with a connecting rod.

## 1. Introduction

Hands are very important in human life, helping people to complete various complex movements and bringing a variety of experiences to life. Amputations due to accidents can adversely affect a patient’s quality of life, preventing them from performing the necessary movements of daily life [1,2,3]. While recent technological advancements have improved the performance of prosthetic hands and made devices increasingly flexible, the question of dexterity versus weight, form factor, and equipment cost of the prosthetic hand remains [4,5].

Actuators for prosthetic hands can be classified into electric, pneumatic, hydraulic, shape memory alloy (SMA), and twisted and curled polymer muscle (TCPM). Electric actuators are the most popular, using different motors with a wide power range, since the motor can obtain the highest output force. Prosthetic hands are more specific applications than robotic hands, with more constraints [6,7,8]. Prosthetics all have to be lightweight, portable, comfortable, and they require a high grip strength to be adopted by the user. The second is the pneumatic actuator, where the air source can be installed in the manipulator. Attempts have been made to use small pneumatic actuators to improve portability, enabling flexible or lightweight designs. Hydraulic actuation is uncommon in prosthetic hands. Flexible actuators such as SMAs and TCPMs are infrequently used, and these actuators have some disadvantages, such as low force and low bandwidth. Addressing these issues could allow these lightweight, inexpensive drivers to improve the design of future prosthetic hands, which seems challenging for all prosthetics [9,10,11,12].

The current complexity of robotic hands limits their development within specialized applications, and under-actuation appears to be one of the promising avenues for prosthetic applications [13,14]. Many researchers have adopted synergistic approaches and adaptive systems to develop affordable, compact prostheses by using differential mechanisms to reduce the number of actuators required to control multiple fingers simultaneously. However, due to different differential structure characteristics, it is difficult to guarantee the weight, flexibility, robustness, controllability, and maintenance cost of the prosthetic hand [15,16,17].

In order to reduce the weight of the prosthesis and increase the grip strength, the motor, as the driver, is one of the topics that need to be studied. There is a desire to simplify the design while maintaining flexibility and control in order to help patients achieve independent living [18]. Under the strict weight limit, it is very important to explore lightweight solutions. The transmission structure, actuator, and controller of the prosthetic hand can reduce its weight through emerging technologies. As the driver of the device, there is currently no good device that is widely used in prosthetics [19,20,21,22,23].

For the purpose of assisting patients with hand function deficiency and their daily life, an ultrasonic rotary motor is designed, and the driving force of the motor is used to realize the flexion, extension, and swing of the fingers, so as to provide help in the daily life of patients. Taking advantage of its advantages of small size, light weight, and fast response time, it can make up for the shortcomings of traditional motors in the field of rehabilitation engineering, provide assistance for patients’ daily life, improve the quality of the living environment, and relieve the pressure caused by the condition. It is also an attempt to apply ultrasonic motors in biomedicine to improve the performance of the prosthetic hand with a connecting rod on the driving device.

An ultrasonic motor is a new type of driver that utilizes the inverse piezoelectric effect of piezoelectric materials [24,25]. Because of its simple structure, high precision, lack of electromagnetic interference, and fast response time, it is widely used in precision machining, biomedical, semiconductor processing and manufacturing, and other technical fields. In recent decades, various ultrasonic motors have been developed including the traveling wave type, standing wave type, and mixed mode type [26,27]. Due to its simple structure, ultrasonic motors have been developed in many studies [28,29,30,31].

The analysis of the elastic body is a research topic that cannot be ignored in the process of ultrasonic motor design and analysis. ANSYS finite element software is used to simulate the actual vibration mode of the stator under fixed constraints. By observing its vibration mode and selecting the available vibration array and vibration frequency according to the driving mode, the harmonic response vibration amplitude and transient motion trajectory of the particles on the surface of the elastomer-driven foot can be calculated at this frequency [32,33,34,35,36]. It can be judged whether its movement displacement meets the requirements of the driving structure stroke, which provides convenience for the structural sizing design of the ultrasonic motor.

To address the issue of “complex structure, slow response, low positioning accuracy, and exceptional volume” [37,38,39,40,41] in existing prosthetic hands, we conducted the research in this article:

(1) Configuration design: Starting from the structural analysis of the prosthetic hand with a connecting rod, the shortcomings and deficiencies of the current motor drive are found. Considering the influence of the traditional motor on the structural limitations, a new ultrasonic motor is designed to optimize the performance of the prosthetic hand with a connecting rod, so as to ensure the flexibility, accuracy, fast response, and power density of the prosthetic hand.
(2) Parameter simulation: We selected the longitudinal vibration piezoelectric metal composite method to design the vibrator, design and simulate the longitudinal vibration system, establish its basic design distribution and structural size, establish a finite element model for the vibrator system, analyze and determine the modal longitudinal vibration frequency, and determine the harmonic response amplitude and maximum displacement of the transient driving the foot surface.
(3) Structural finalization: The fixing method of the vibration system is determined. Under the action of sinusoidal voltage, the motion of the ultrasonic vibrator is calculated to meet the input shaft speed required by the prosthetic hand, and the overall structure of the ultrasonic motor is determined, which makes the application of the ultrasonic motor in the prosthetic hand with a connecting rod possible. This will also be an attempt to explore the application of ultrasonic motors in the direction of prosthetic robots, while sufficiently preparing theoretically for the production of prosthetic drives.

## 2. Materials and Methods

### 2.1. Design of Prosthetic Hand and Motor Structure

**Figure 1:**
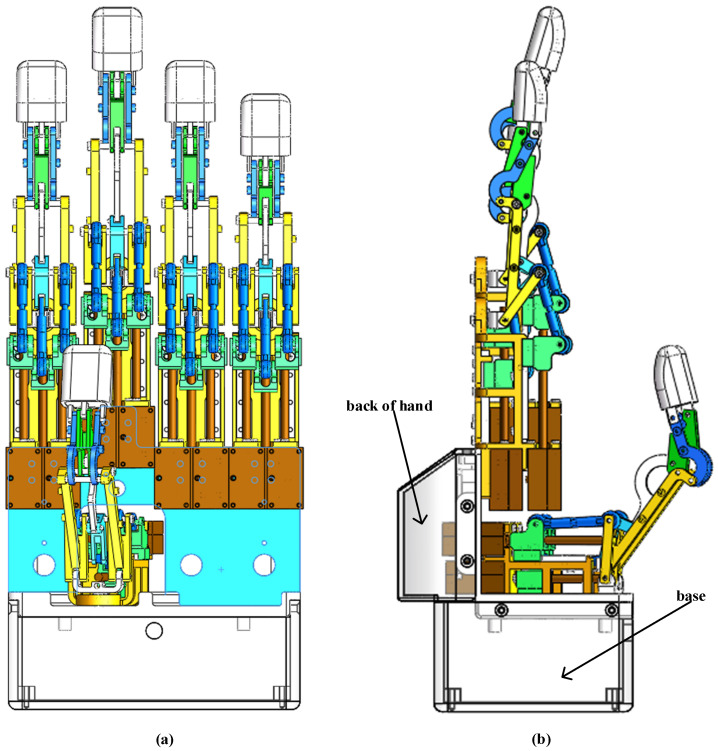
Dexterous prosthetic hand model. (**a**) Assembly front view; (**b**) assembly side view.

The link drive mechanism is often used in hand-function robotic devices. Through a structure that stimulates the movement of the joint in the required direction, the fingers can be driven to achieve flexion and extension movements. In the process of motion, the flexibility, controllability, portability, comfort, and safety of the robot are particularly important, and its design requires a compact structure, low production cost and low maintenance cost. This paper mainly focuses on the problems and improvement methods of the prosthetic hand driver. Figure 1 shows the overall assembly diagram of the prosthetic hand with a connecting rod.

**Figure 2:**
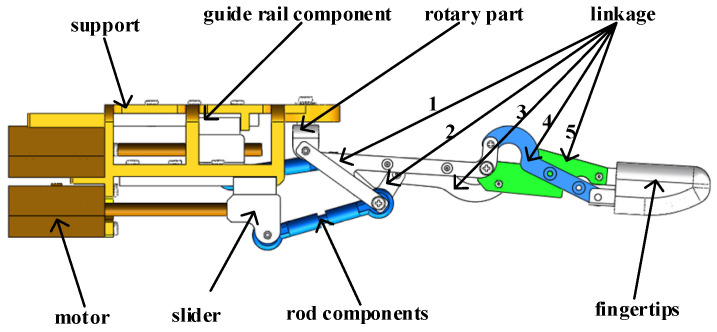
Assembly drawing of single finger structure.

Each finger is mainly composed of an ultrasonic motor, a support, a guide rail, a slider, a pull rod, a rotating part, a connecting rod structure, and a fingertip. Due to its working principle, the traditional electromagnetic drive is large in size, heavy in weight, and slow in response time. The output torque is small and accompanied by a certain amount of noise, which will lead to psychological pressure and life burden for the wearer. In order to solve the above problems, a driver with ultrasonic vibration as the power source will be designed, which has the advantages of small size, small mass, simple structure, accurate positioning, high power density, and fast response time. It is enough to improve the driving performance of the prosthetic hand with a connecting rod on the device. Figure 2 is the assembly diagram of the assembled driver assembled in the structure of a single finger.

**Figure 3:**
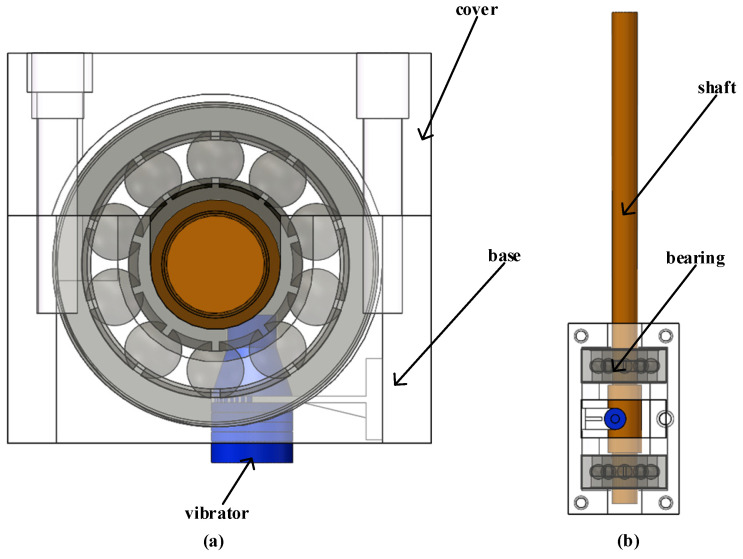
Structural diagram of an ultrasonic driver. (**a**) Assembly front view; (**b**) Assembly side view.

As shown in Figure 3, the two ends of the stepped shaft are positioned by bearings, the preload provided by the upper cover acts on the stepped shaft, and the pressure is transmitted to the stepped shaft. The vibrator’s drive foot of the ultrasonic driver is in contact with the shaft. Under the influence of pressure, the vibrator generates friction with the output shaft during the vibration process, and then drives the shaft to rotate. As shown in Figure 3, the rotary motion of the shaft drives the slider to move in the direction of the specified guide rail, thereby driving the pull rod and connecting rod and fingertip structure to move to complete the flexion and extension of the finger. Finger swinging can also be accomplished by controlling the motor. Figure 3 is a structural diagram of an ultrasonic driver.

### 2.2. Structural Design of Longitudinal Vibration System

**Figure 4:**
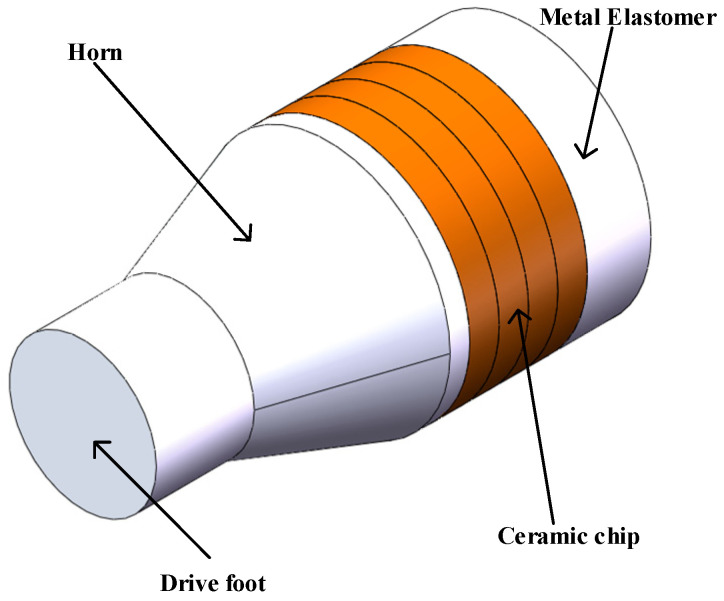
Vibrator structure diagram.

In this section, an ultrasonic vibration system will be designed for the motor-driven structure of a prosthetic hand. The structure of the longitudinal vibration composite piezoelectric vibrator is shown in Figure 4. The structure of the vibrator is symmetrical and consists of a driving foot, a horn, a ceramic sheet, and a metal elastic body. The circular driving foot is located at the front end of the horn, and the function of the horn is amplitude amplification. Transform the vibration of the transducer end into a specific large-amplitude mechanical vibration for a specific vibration mode of the vibration system. The four piezoelectric ceramic sheets are, respectively, attached to the middle of the metal elastomer, and the working mode of the piezoelectric ceramic is d31. In order to realize the small size of the vibrator, epoxy resin glue is used to stick the ceramic sheet onto the metal base.

### 2.3. Resonant Frequency Equation

**Figure 5:**
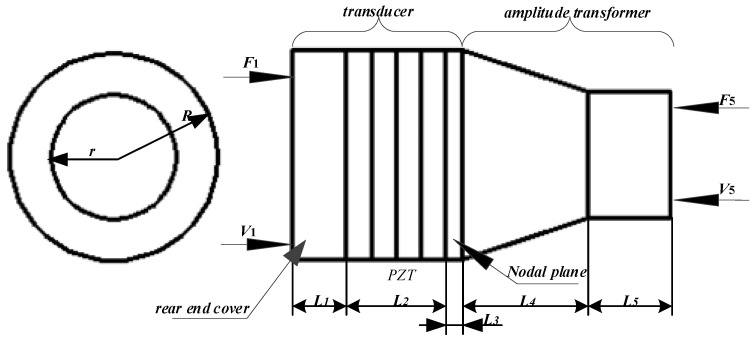
Vibrator structure size diagram.

The characteristic equation of the four-terminal network transmission on the left side of the nodal plane in Figure 5 is

(1) $$\left[\begin{array}{l} V_{3}\\ F_{3} \end{array}\right]=\left[\begin{array}{l} a_{11}\;\;\;a_{12}\\ a_{21}\;\;\;a_{22} \end{array}\right]\left[\begin{array}{l} V_{1}\\ F_{1} \end{array}\right]$$

In the formula:

*V_i_*—Vibration velocity parameters of rods at the end surface;
*F_i_*—Force parameters of rods at the end surface;
$\left[\begin{array}{l} a_{11}\;\;\;a_{12}\\ a_{21}\;\;\;a_{22} \end{array}\right]$—transfer matrix.

The transfer matrix is

(2) $$\left[\begin{array}{l} a_{11}\;\;\;a_{12}\\ a_{21}\;\;\;a_{22} \end{array}\right]=\left[\begin{array}{l} a^{3}{}_{11}\;\;\;a^{3}{}_{12}\\ a^{3}{}_{21}\;\;\;a^{3}{}_{22} \end{array}\right]\left[\begin{array}{l} a^{2}{}_{11}\;\;\;a^{2}{}_{12}\\ a^{2}{}_{21}\;\;\;a^{2}{}_{22} \end{array}\right]\left[\begin{array}{l} a^{1}{}_{11}\;\;\;a^{1}{}_{12}\\ a^{1}{}_{21}\;\;\;a^{1}{}_{22} \end{array}\right]$$

The vibration system is generally composed of multiple rods with different cross-sectional shapes that are connected, and commonly used shapes include equal cross-section rods, cones, etc.

The transfer matrix parameters of the uniform cross-section rod can be simplified as follows [25,26,27]:

(3) $$\left\{\begin{array}{l} a_{11}=\cos kL\\ a_{21}=-j\rho cS\sin kL \end{array}\right.\;\;\;\;\;\;\;\;\;\;\;\;\;\;\left.\begin{array}{l} a_{12}=\frac{-j\sin kL}{\rho cS}\\ a_{22}=\cos kL \end{array}\right\}$$

The transfer matrix parameters of a conical shaped rod can be simplified as

(4) $$\left\{\begin{array}{l} a_{11}=\frac{-\left(\alpha /k\right)\sin kL+\cos kL}{1-\alpha L}\\ a_{21}=\frac{\rho cS_{1}}{jk}\left\{\left[k\left(1-\alpha L\right)+\frac{\alpha^{2}}{k}\right]\sin kL-\alpha^{2}L\cos kL\right\} \end{array}\right.\;\;\;\;\;\;\;\;\left.\begin{array}{l} a_{12}=\frac{-j\sin kL}{\rho cS_{1}(1-\alpha L)}\\ a_{22}=(1-\alpha L)\cos kL+\frac{\alpha }{k}\sin kL \end{array}\right\}$$

According to the motion state and force condition of the left part of the nodal plane, the parameters at both ends are determined as *V*_3_ = 0 and *F*_1_ = 0, and *a*_11_ = 0 is obtained by substituting into Formula (1). Substituting into the parameters of the four-terminal network transfer matrix of the equal-section rod, the resonance frequency equation can be obtained as

(5) $$\frac{Z_{1}}{Z_{2}}\tan k_{2}L_{2}\tan k_{1}L_{1}+\frac{Z_{1}}{Z_{3}}\tan k_{1}L_{1}\tan k_{3}L_{3}+\frac{Z_{2}}{Z_{3}}\tan k_{3}L_{3}\tan k_{2}L_{2}=1$$

In the formula:

k—wave number, ki=ωici, $\omega_{i}$ is the angular frequency of ultrasonic vibration, i = 1, 2, 3;
$L_{i}$—length of each rod, *i* = 1, 2, 3;
$Z_{i}$—Equivalent impedance, $Z_{i}=\rho_{i}c_{i}S_{i}$, $\rho_{i}$ is the material density, $c_{i}$ is the longitudinal propagation velocity of ultrasonic waves;
$S_{i}$ is the cross-sectional area, *i* = 1, 2, 3.

For the right side of the nodal plane, that is, the four-terminal network transmission, the characteristic equation of the primary and secondary composite horns is

(6) $$\left[\begin{array}{l} V_{5}\\ F_{5} \end{array}\right]=\left[\begin{array}{l} b_{11}\;\;\;b_{12}\\ b_{21}\;\;\;b_{22} \end{array}\right]\left[\begin{array}{l} V_{4}\\ F_{4} \end{array}\right]$$

The transfer matrix is

(7) $$\left[\begin{array}{l} b_{11}b_{12}\\ b_{21}b_{22} \end{array}\right]=\left[\begin{array}{l} b^{5}{}_{11}\;\;\;b^{5}{}_{12}\\ b^{5}{}_{21}\;\;\;b^{5}{}_{22} \end{array}\right]\left[\begin{array}{l} b^{4}{}_{11}\;\;\;b^{4}{}_{12}\\ b^{4}{}_{21}\;\;\;b^{4}{}_{22} \end{array}\right]$$

According to the motion state and force condition of the right part of the nodal plane, the parameters of both ends are determined as *V*_4_ = 0 and *F*_5_ = 0. Substitute into Formula (6) to get *b*_22_ = 0. Substituting into the transmission matrix parameters of the four-terminal network of equal-section rods and conical rods, the resonance frequency equation can be obtained as

(8) $$\left(1-L_{4}\alpha_{4}+\frac{\alpha_{4}\tan(k_{4}L_{4})}{k_{4}}\right)=-z_{5}\tan(k_{5}L_{5})\frac{\tan(k_{4}L_{4})}{z_{4}(L_{4}\alpha_{4}-1)}$$

In the formula:

k—wave number, ki=ωici, (i = 4, 5);
$\omega_{i}$ is the angular frequency of ultrasonic vibration, (i = 4, 5);
$L_{i}$—length of each rod, (*i* = 4, 5);
$N_{j}$—Section reduction rate, $N_{j}=\sqrt{\frac{S_{j}{}^{1}}{S_{j}{}^{2}}}=\frac{R_{j}{}^{1}}{R_{j}{}^{2}}$, (*j* = 4);
$\alpha_{j}$—Taper factor, $\alpha_{j}=\left(N_{j}-1\right)/\left(N_{j}L_{j}\right)$, (*j* = 4);
$Z_{i}$—Equivalent impedance, $Z_{i}=\rho_{i}c_{i}S_{i}$, $\rho_{i}$ is the material density, $c_{i}$ is the longitudinal propagation velocity of ultrasonic waves;
$S_{i}$ is the cross-sectional area, *i* = 4, 5.

### 2.4. Structure Size

For the entire vibration system, we want the ultrasonic vibration wave to propagate forward as far as possible. This requires that the rear end of the vibration system is heavier than the front end, that is, the rear end is denser than the front end. Taking all factors into consideration, stainless steel 2Cr13 was selected for the rear end cover, PZT-8 was selected for the piezoelectric ceramic sheet, and 7A04, a lighter duralumin alloy, was used for the rest. The relevant parameters of the three materials are presented in Table 1.

As shown in Figure 4, the rear end cover and piezoelectric ceramic are connected with the transducer through hard aluminum alloy. If the design and calculation of the *L*_1_ and *L*_2_ sections are completely based on the material properties of the rear end cover or piezoelectric ceramics, there must be a large error. Therefore, this paper adopts the equivalent area method for calculation, which has the following conversion relationship:

(9) $$\left\{\begin{array}{l} \rho_{\mathrm{rear}\;\mathrm{end}\;\mathrm{cover}}=\frac{\rho_{1}S_{1}+\rho_{3}S_{3}}{S_{13}}\\ \rho_{\mathrm{ceramics}}=\frac{\rho_{2}S_{2}+\rho_{3}S_{3}}{S_{23}}\\ C_{\mathrm{rear}\;\mathrm{end}\;\mathrm{cover}}=\frac{C_{1}S_{1}+C_{3}S_{3}}{S_{13}}\\ C_{\mathrm{ceramics}}=\frac{C_{2}S_{2}+C_{3}S_{3}}{S_{23}} \end{array}\right.$$

In the formula:

$S_{13}$—The total cross-sectional area of the connecting section between the rear end cover and the hard aluminum alloy, $S_{13}=S_{1}+S_{3}$, $S_{1}$ is the cross-sectional area of the rear end cap, $S_{3}$ is the cross-sectional area of duralumin;
$S_{23}$—The total cross-sectional area of the connecting section between the piezoelectric ceramic sheet and the hard aluminum alloy, $S_{23}=S_{2}+S_{3}$, $S_{2}$ is the cross-sectional area of the piezoelectric ceramic sheet.

The PZT-8 selected in this paper is a circular piezoelectric ceramic sheet polarized along the thickness direction. Its inner diameter is φ 0.5 mm, outer diameter is φ 1.25 mm, and thickness is 0.3 mm. Here, the diameter and length of each section of the vibrator are taken as shown in Table 2, where R represents the outer diameter or large-end diameter, and r represents the inner diameter or small-end diameter. We substituted the relevant parameters into the above two resonance frequency Equations (3) and (6), and calculated using Matlab R2017a programming, *L*_1_ = 0.65 mm, *L*_5_ = 1.0 mm

## 3. Results and Discussion

### 3.1. Simulation Analysis of Vibrator

**Figure 6:**
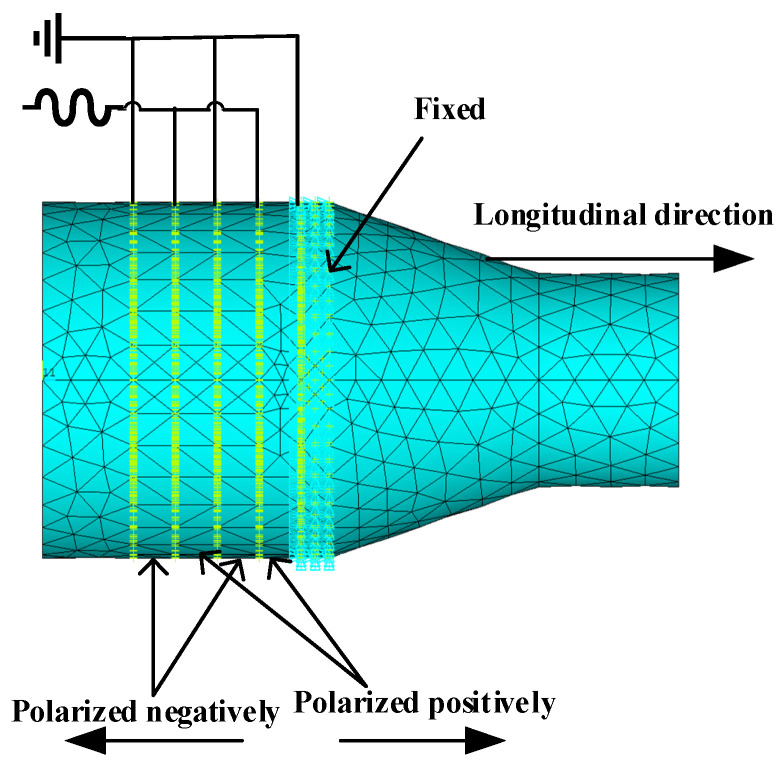
Polarization mode and constraint settings of the vibrator.

A finite element simulation of the ultrasonic vibration system performed using APDL in the ANSYS environment builds an overall model of the entire ultrasonic vibration system with a command flow and analyzes the finite element model of the whole vibration system. Meshing a vibration system using SOLID227 elements, the system divides the grid model with a qualified quality and sets the conditions of the model before analysis. The voltage distribution position, polarization direction, and fixing method are shown in Figure 6.

### 3.2. Modal Analysis and Harmonic Analysis

We performed modal response analysis on the vibration system, imposed constraints during operation, and obtained the different modal formations of the system and their corresponding resonance frequencies to pave the way for future dynamic analysis of the longitudinal vibration system; at the same time, it guides the application of conditions in the actual test. We solved its modes using command flow in ANSYS 19.2; the selection range is 20 KHz–300 KHz in order to extract the first six order frequencies of the system, as shown in Table 3.

**Figure 7:**
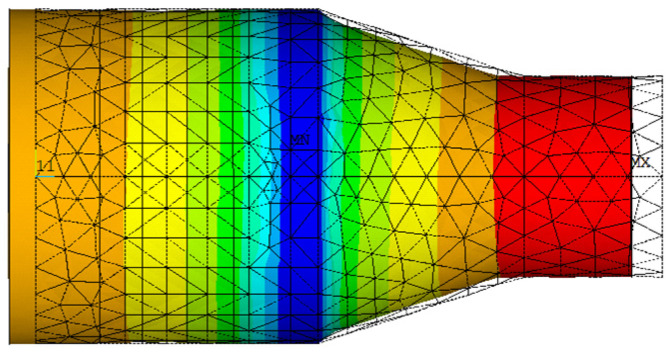
Longitudinal mode of the vibrator at 279.6 kHz.

We extracted the longitudinal vibration mode corresponding to the frequency of 279,633 Hz of the vibration system. The end of the horn vibrates along the longitudinal direction, as shown in Figure 7.

It can be seen from the figure that the longitudinal vibration frequency of the structure is 279.6 KHz. When the system is subjected to an electrical signal of this frequency, the vibration form of the system is as shown in the figure above, and the end has the largest longitudinal amplitude. This frequency is the resonance frequency of the ultrasonic system during operation. The above constraints are applied to the system, a sinusoidal voltage of 279,633 ± 5 Hz is given to both ends of the ceramic plate, and the effective value of its voltage is set at 200 V. The harmonic response of the system during longitudinal vibration can be obtained, so as to obtain the maximum amplitude of its end resonance and the resonance amplitude in the XY direction.

**Figure 8:**
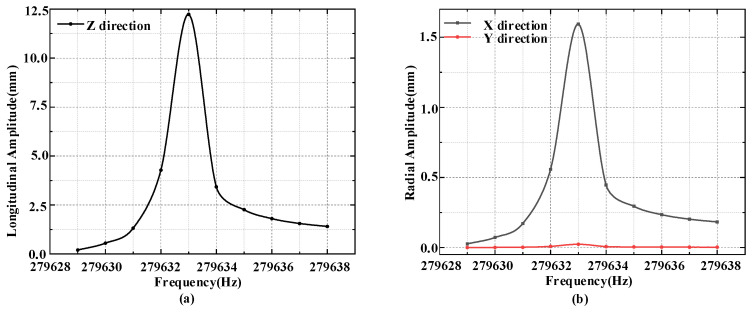
Monic response of the vibration system: (**a**) Longitudinal amplitude vs. frequency of the vibrator; (**b**) Radial amplitude versus frequency in X and Y directions.

The results are shown in Figure 8. It can be seen that the maximum axial amplitude of the resonance response at the end of the vibrator system is 12.23 mm, which is much larger than the maximum radial resonance amplitude of 1.5 mm.

### 3.3. Transient Analysis and Output Shaft Speed

In order to accurately reflect the actual working conditions of the ultrasonic vibration system, the transient analysis of the vibrator is carried out to obtain the motion path of the particle over time. In transient analysis, the frequency of the alternating voltage that is applied to the electrodes is 279.6 kHz, the effective value is 200 V, the phase difference is 90 degrees, and the damping factor is 0.003. In order to obtain the vibration characteristics of the vibrator, the motion path curve of the particles on the surface of the driving foot is extracted after analysis.

**Figure 9:**
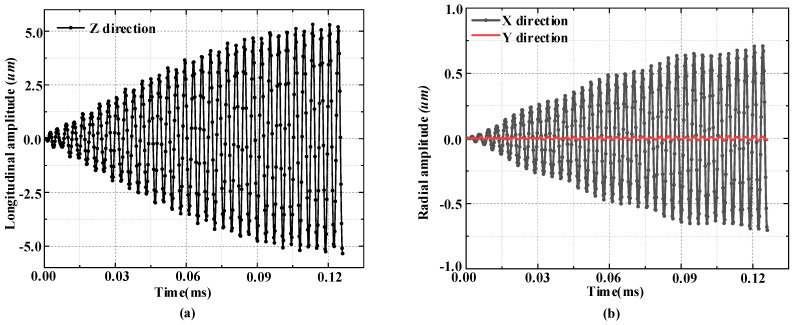
Transient analysis of a vibrating system driven by sinusoidal voltages with a phase difference of 90°. (**a**) Longitudinal amplitude of the vibrator; (**b**) Radial amplitude in X and Y directions.

As shown in Figure 9a, in the results of the transient analysis of the ultrasonic vibration system under sine wave excitation, the axial vibration amplitude of the contact point of the horn end is much larger than the radial amplitude, and the maximum longitudinal amplitude at the extracted contact point is 5.34 μm. Figure 9b shows the radial amplitudes of the driving foot in the X and Y directions. It can be seen that the corresponding lateral amplitude during longitudinal vibration is small, and the maximum amplitude can only be about 0.7 μm. That is, a larger longitudinal amplitude and a certain radial amplitude are generated.

From the above analysis, it can be seen that under sinusoidal voltage excitation of 200 V rms, the vibration system can achieve a longitudinal amplitude of about 5.3 μm. It can provide a speed of about 2.6 m/s for the output shaft, which fully meets the linear movement speed requirements.

### 3.4. Discussion

This article starts with a structural analysis of a prosthetic hand with a connecting rod and identifies its shortcomings and the shortcomings of the current motor drive. Considering the influence on the traditional motor of the structural limitations, a new ultrasonic motor is designed to optimize the performance of the prosthetic hand with a connecting rod, so as to ensure the flexibility, accuracy, fast response, and power density of the prosthetic hand.

Through the above structural design, experimental analysis, and calculation results, it can be seen that the designed ultrasonic motor can be used as the driver of the prosthetic hand. In order to reduce the structural weight, most prostheses currently use under-actuated methods. The use of a differential structure to reduce the number of drivers makes the overall structure of the prosthesis complex, and the driving effect is also affected. The application of ultrasonic motors in biomedicine has already begun, and here, it is applied to the field of prosthetic drives to reduce the weight of prosthetic hands and improve the performance of prostheses. In the future, prosthetics will develop in the direction of light weight, dexterity, and intelligence. As a transmission structure, whether it is a connecting rod transmission or a rope transmission, the requirement for the driver will be a micromotor. Ultrasonic motors can fully comply with the design of a miniature motor structure. While ensuring a fast response and positioning accuracy, it reduces weight and provides significant power density to ensure the normal operation of the prosthesis. This will be a work worth studying.

However, the current research also has certain limitations. At present, the driving power supply for power ultrasound is still relatively large, which is a major limitation for the application of this article’s achievements. We hope that with the development of technology, better driving technologies can be developed, so as to promote the prosthetic hand solution proposed in this article to the market.

## 4. Conclusions

In response to the shortcomings of complex structure, slow response, low positioning accuracy, and large volume of current prosthetic hands, this paper designs an ultrasonic-driven prosthetic hand structure. By designing a longitudinal vibration linear motor for a prosthetic hand, better driving characteristics can be achieved.

This article conducts modal analysis, harmonic response analysis, and transient analysis simulation on the stator of the designed ultrasonic motor. The feasible design of the structural dimensions of the prosthetic ultrasonic actuator of the connecting rod has been achieved, providing parameter guidance for its design and manufacturing. Through the research in this article, the structure of the ultrasonic motor has been determined, making the application of the ultrasonic motor in a dexterous prosthetic hand possible, and making sufficient theoretical preparations for the production of prosthetic limb drivers.

## Figures and Tables

**Table 1:** Material parameters.

Material	Density	Wave Velocity
2Cr13	$\rho_{1}=7.75\times 10^{3}\;kg/m^{3}$	$c_{1}=5200\;m/s$
PZT-8	$\rho_{2}=7.5\times 10^{3}\;kg/m^{3}$	$c_{2}=3200\;m/s$
7A04	$\rho_{3}=2.7\times 10^{3}\;kg/m^{3}$	$c_{3}=5100\;m/s$

**Table 2:** The diameter and length of each section of the vibrator.

r (mm)	R (mm)	*L*_1_ (mm)	*L*_2_ (mm)	*L*_3_ (mm)	*L*_4_ (mm)	*L*_5_ (mm)
0.75	1.25	0.65	1.2	0.2	1.5	1

**Table 3:** The first 6 order frequencies of the system.

Order	1	2	3	4	5	6
Frequency/Hz	147,319	153,129	185,826	187,821	261,215	279,633

## Data Availability

The data presented in this study are available on request from the corresponding author.
